# A Novel Prostate-Specific Membrane-Antigen (PSMA) Targeted Micelle-Encapsulating Wogonin Inhibits Prostate Cancer Cell Proliferation via Inducing Intrinsic Apoptotic Pathway

**DOI:** 10.3390/ijms17050676

**Published:** 2016-05-17

**Authors:** Hailong Zhang, Xiaogang Liu, Fengbo Wu, Feifei Qin, Ping Feng, Ting Xu, Xiang Li, Li Yang

**Affiliations:** 1 State Key Laboratory of Biotherapy, West China Hospital, West China Medical School, Sichuan University, Chengdu 610041, China; hailong6891@163.com (H.Z.); sklb_qff@sina.com (F.Q.); 2 Department of Gastroenterology, Hospital of the University of Electronic Science and Technology of China and Sichuan Provincial People’s Hospital, Chengdu 610041, China; lxg_sph@163.com; 3 Department of Pharmacy, West China Hospital, West China Medical School, Sichuan University, Chengdu 610041, China; wufengbo_@163.com (F.W.); xt_wch@163.com (T.X.); 4 Institute of Clinical Trials, West China Hospital, West China Medical School, Sichuan University, Chengdu 610041, China; 5 Department of Urology, West China Hospital, West China Medical School, Sichuan University, Chengdu 610041, China

**Keywords:** prostate specific membrane-antigen, wogonin, prostate cancer, apoptosis

## Abstract

Prostate cancer (PCa) is a malignant tumor for which there are no effective treatment strategies. In this study, we developed a targeted strategy for prostate-specific membrane-antigen (PSMA)-positive PCa *in vitro* based on 2-(3-((*S*)-5-amino-1-carboxypentyl)ureido) pentanedioic acid (ACUPA) modified polyethylene glycol (PEG)-Cholesterol micelles containing wogonin (WOG), which was named ACUPA-M-WOG. ACUPA-M-WOG was conventionally prepared using a self-assembling method, which produced stable particle size and ζ potential. Moreover, ACUPA-M-WOG showed good drug encapsulating capacity and drug release profiles. Fluorescence activated cell sorting (FACS) results suggested that ACUPA modified PEG-Cholesterol micelles could effectively enhance the drug uptake on PSMA(+) PCa cells, and the cytotoxicity of ACUPA-M-WOG was stronger than other controls according to *in vitro* cellular proliferation and apoptosis assays, separately through methyl thiazolyl tetrazolium (MTT) and Annexin V/Propidium Iodide (PI) staining. Finally, the molecular mechanisms of ACUPA-M-WOG’s effects on human PSMA(+) PCa were investigated, and were mainly the intrinsic or extrinsic apoptosis signaling pathways. The Western blot results suggested that ACUPA-M-WOG could enhance the WOG-induced apoptosis, which was mainly via the intrinsic signaling pathway rather than the extrinsic signaling pathway. In conclusion, ACUPA-M-WOG was successfully developed for WOG-selective delivery to PSMA(+) PCa cells and had stronger inhibition than free drugs, which might make it an effective strategy for PSMA(+) PCa.

## 1. Introduction

**Figure 1:**
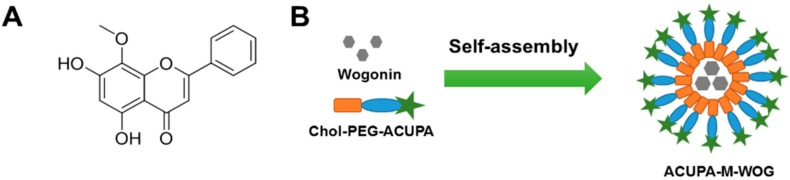
(**A**) The chemical structure of wogonin; (**B**) Schematic representation of self-assembled 2-(3-((*S*)-5-amino-1-carboxypentyl)ureido)pentanedioic acid (ACUPA) modified polyethylene glycol (PEG)-Cholesterol micelles containing wogonin (WOG) (ACUPA-M-WOG).

As a major cause of cancer-related death in male patients in Europe and USA, prostate cancer (PCa) is a great burden for both patients and public medical systems. In the early stage of PCa, surgery, radiation, and androgen deprivation therapy (ADT) are the standard therapeutic procedures and relatively effective for localized PCa [1]. However, after a period of ADT, PCa can transform to castration-resistant prostate cancer (CRPC), with high potency of metastasis and development. Nowadays, chemotherapy is the most common used approach to combat CRPC, despite its unsatisfactory therapeutic efficacy due to lack of target selection and unspecific toxicity [2]. Although paclitaxel-based chemotherapy is effective in some clinical practices, the poor hydrophilicity and bioavailibility of paclitaxel markedly limit its maximum therapeutic potency [3]. In the last few decades, encapsulation of paclitaxel into nano-formulations has achieved reasonable success as a pharmaceutical strategy. In our previous studies, encapsulation of paclitaxel into a modified PEGylated long-circulating liposome could increase its bioactivity and prolong the drug half-life [4,5,6]. Recently, as one of the main bioactive natural products of *Scutellaria baicalensis*, wogonin (Figure 1A) has been found to exert potent activities against a variety of cancers. Moreover, several reports indicated that wogonin significantly inhibited cellular proliferation and resulted in apoptosis through reactive oxygen species (ROS) release in many cancer cells [7], e.g., SKOV3, LNCaP, A2780, HCT-116 and Hela cells [8].

Prostate-specific membrane-antigen (PSMA) is a member of type II transmembrane glycosylated protein with a molecular weight of about 100 kDa. It is mainly composed of three functional domains: a C-terminal domain, a protease domain and an apical domain [9,10,11,12,13]. The expression level of PSMA is obviously up-regulated in prostate cancer cells than normal prostate issue or endothelial cells. The genomics researches and pathological analysis have proved that PSMA is one of the most increased expression proteins in PCa, and the PSMA expression level shows a positive correlation with the potency of tumor progress or metastasis [14,15,16,17,18,19]. In recent years, several strategies have been developed to achieve targeting capacity for PCa in PSMA targeted prodrugs or nanomedicines, such as peptides, RNA aptamers and monoclonal antibodies (mAb). The phage display technology is used to screen and identify binding peptides for PSMA, and the results suggest that KYLAYPDSVHIW and WQPDTAHHWATL can bind to PSMA with high affinity and inhibit its glutamate carboxypeptidase activity [20,21,22]. Similarly, Lupold *et al.* [23,24,25,26,27] discovered that some RNA aptamers can efficiently recognize PSMA and inhibit the enzymatic activity. Moreover, some protein drugs, such as anti-PSMA mAbs, single-chain variable fragment (scFv) and soluble receptors have been used to target PCa [28,29,30,31]. For example, Indium-111 radio-labeled anti-PSMA mAb (mAb 7E11) has been approved by Food and Drug Administration (FDA) for the radiographic test of PCa [30,32,33,34,35,36] Some mAbs-conjugated immunotoxins or nanoparticles as PCa-targeted agents have been tested in clinical trials [37,38,39,40,41,42,43,44,45,46,47,48,49].

2-[3-(1,3-Dicarboxypropyl)ureido]pentanedioic acid (DUPA) is one of the highest-affinity small molecular ligands of PSMA [50,51]. After binding to PSMA, DUPA can be immediately endocytosed into clathrin-coated pits, and PSMA can release DUPA into cytoplasm and then return to the cell membrane. Recently, Post *et al.* [50,52] report a radio-labeled conjugate of DUPA and ^99m^*T*c derivative for selecting patients suitable for PSMA-targeted therapy and diagnosing PCa progress or recurrence. Some subsequent reports show DUPA-linked cytotoxins can efficiently inhibit tumor proliferation both *in vitro* and *in vivo* with moderate to good specificity. As a chemical mimic of DUPA, 2-(3-((*S*)-5-amino-1-carboxypentyl)ureido)pentanedioic acid (ACUPA) also shows satisfactory targeted efficiency at the protein, cell and whole animal levels [51,52,53,54,55,56].

We have previously reported that peptide-modified polyethylene glycol (PEG)-Cholesterol (Chol) (PEG-Chol) micelles can specifically recognize fibroblast growth factor receptor (FGFR), enhance paclitaxel delivery to cancer cells and induce cytotoxicity in FGFR overexpressed cancer cells [57,58,59,60]. Although several aptamer- or mAb-modified nano-formulations have been reported and their PCa-targeted abilities have been evaluated, to our knowledge, only a few studies about the preparation and evaluation of ACUPA based drug delivery system have been previously conducted. The detailed molecular mechanisms and targeted and anticancer ability remain elusive. The main aim of this study is to validate the targeted delivery of antitumor natural product wogonin (WOG) to PCa by ACUPA modified PEG-Chol (ACUPA-PEG-Chol) micelles (Figure 1B), to verify its anti-tumor efficiency, and to observe the molecular mechanism in cell lines. The effects of apoptosis inhibitor on the cellular proliferation of LNCaP cells are evaluated to clarify the action mechanism of WOG loaded micelles. Finally, the apoptotic related proteins are detected by Western blot, which is measured to elucidate the action mechanisms and to determine the potential apoptosis pathway of the WOG loaded micelles.

## 2. Results and Discussion

### 2.1. Characterization of 2-(3-((S)-5-Amino-1-carboxypentyl)ureido)pentanedioic Acid (ACUPA) Modified Polyethylene Glycol (PEG)-Cholesterol (Chol) (ACUPA-PEG-Chol) and Wogonin (WOG) Loaded Micelles

**Figure 2:**
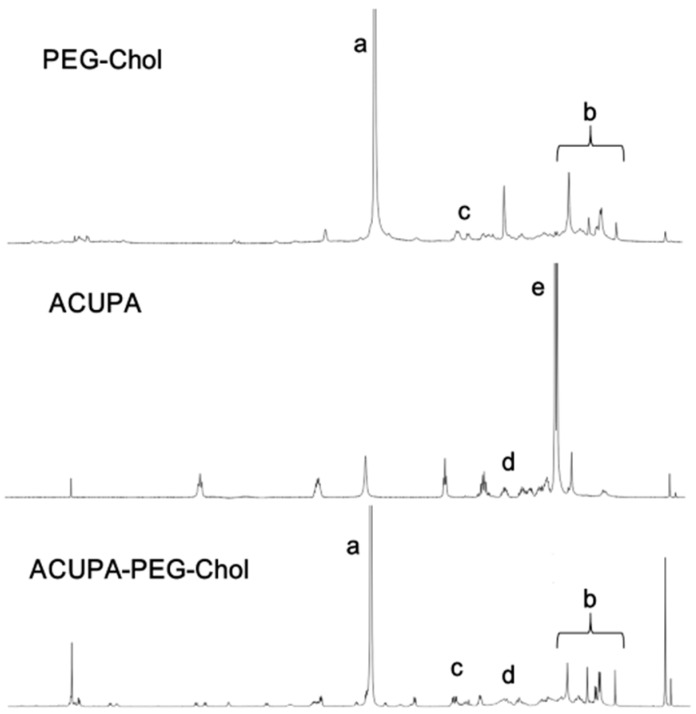
The ^1^H NMR spectrum of 2-(3-((*S*)-5-amino-1-carboxypentyl)ureido)pentanedioic acid (ACUPA) modified (ACUPA-PEG-Chol), ACUPA and polyethylene glycol (PEG)-Cholesterol (Chol) (PEG-Chol).

**Figure 3:**
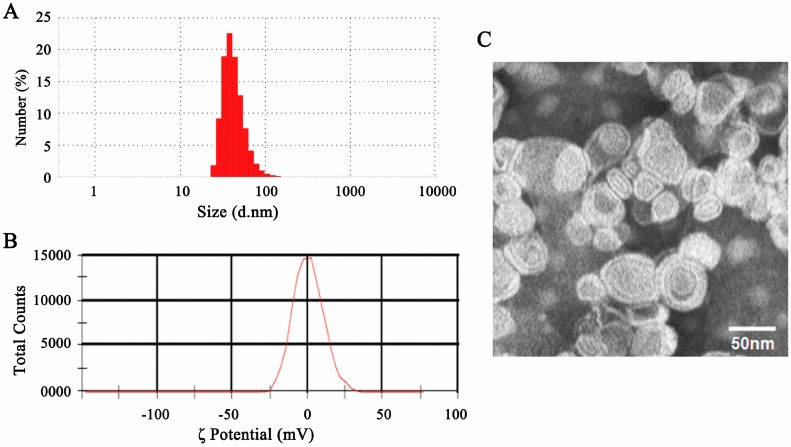
The features of ACUPA-M-WOG. (**A**) Particle size distribution of ACUPA-M-WOG; (**B**) ζ potential of ACUPA-M-WOG; (**C**) TEM image of ACUPA-M-WOG.

The synthesis route of ACUPA-PEG-Chol is referenced in previous reports and some modifications are made [24,30]. Figure 2 presents the ^1^H nuclear magnetic resonance (^1^H NMR) spectrum of ACUPA-PEG-Chol and some intermediates, ACUPA and PEG-Chol, which means ACUPA-PEG-Chol is successfully synthesized. The large peak at about δ 3.5 ppm (a) shows the methylene group in PEG, which contains repeating ethylene glycol units; only PEG-Chol and ACUPA-PEG-Chol exhibit this peak. The multiple peaks from δ 0.6 to 1.2 ppm (b) are assigned to the protons of cholesterol scaffold, and the dd peaks at around δ 2.6 ppm (c) show the methylene proton of the succinyl group. The multiple peaks around δ 2.0 ppm (d) are assigned to the methylene of the glutamate group in the ACUPA fragment, and the adjacent two single peaks at δ 1.40 and 1.38 ppm (e) are assigned to the *tert*-butyl ester group in the ACUPA fragment. These results further show that the conjugate has reacted well and target molecule was successfully synthesized.In general, the preparation of ACUPA-M-WOG is utilized a self-assembly method, and the mean size of wogonin loaded micelles is about 24 ± 5 nm (Figure 3A), with an optimal drug/polymer (*w*/*w*) ratio of 1:19, and optimal drug loading (DL) and entrapped efficiency (EE) of 4.8% ± 0.2% and 95.8% ± 2.6%, respectively. Moreover, the polydisperse index (PDI), and ζ potential of WOG-loaded ACUPA-PEG-Chol micelles are 0.27 ± 0.03, and 0.6 ± 1.4 mV, respectively (Figure 3B). The transmission electron microscopy (TEM) image of ACUPA-M-WOG is demonstrated in Figure 3C, which implies that the ACUPA-M-WOG is nearly spherical in shape and its diameter is about 30 nm. The results of particle size and microscopic structure of micelles tested by TEM prove the stable and homogenous ACUPA-M-WOG aqueous disperse can be achieved by loading WOG into AUPA-PEG-Chol amphiphilic polymeric micelles.

### 2.2. Specific Cellular Uptake of the Micelles

**Figure 4:**
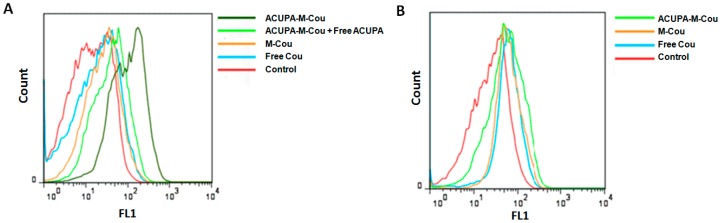
The fluorescence intensities in LNCaP and PC-3 cells treated with ACUPA-M-Cou (coumarine-6 loaded ACUPA modified micelles). (**A**) The fluorescence intensities in LNCaP cells respectively incubated with ACUPA-M-Cou, ACUPA-M-Cou plus free ACUPA, M-Cou (coumarine-6 loaded micelles), free coumarine-6, and PBS. Moreover, the mean fluorescence intensities of each group are quantified; (**B**) The fluorescence intensities in PC-3 cells respectively treated with ACUPA-M-Cou, M-Cou, free coumarine-6, and PBS. Moreover, the mean fluorescence intensities of each group are quantified.

The LNCaP and PC-3 cells are incubated with micelles containing coumarine-6 (Cou), to evaluate whether the increased drug uptake of ACUPA is modified by prostate cancer cells. After incubation of free coumarine-6, M-Cou and ACUPA-M-Cou with or without ACUPA for a certain time interval are washed with PBS twice. Then, the cells are collected and flow cytometry is used to detect the fluorescence, which is derived from coumarine. The result (Figure 4A) shows the fluorescence intensity in LNCaP cells incubated with ACUPA-M-Cou, ACUPA-M-Cou plus free ACUPA, M-Cou, free coumarine-6 and PBS. The mean fluorescence intensity of ACUPA-M-Cou group cells is 4.3-fold stronger (mean fluorescence intensity = 5. 4 *vs.* 23.2, *p* < 0.05) than that of the M-Cou group (Figure 4A); this result suggests that the ACUPA fragment indeed increases the uptake of the micelles in PSMA-positive PCa cells. Moreover, when the free ACUPA is added in the culture beforehand, the uptake increase of the ACUPA-M-Cou group is eliminated, which suggests that the binding of ACUPA to PSMA occurs in a competitive manner. In Figure 4B, on the PSMA negative PC-3 cells, there are no significant differences in mean fluorescence intensity of ACUPA-M-Cou, M-Cou and free Coumarine-6 groups, which further reveals the ability of ACUPA-M-Cou micelles to target PSMA positive PCa cells relies on the ACUPA fragments binding to PSMA.

### 2.3. In Vitro Cytotoxicity and Apoptosis Assay of the Micelles

**Figure 5:**
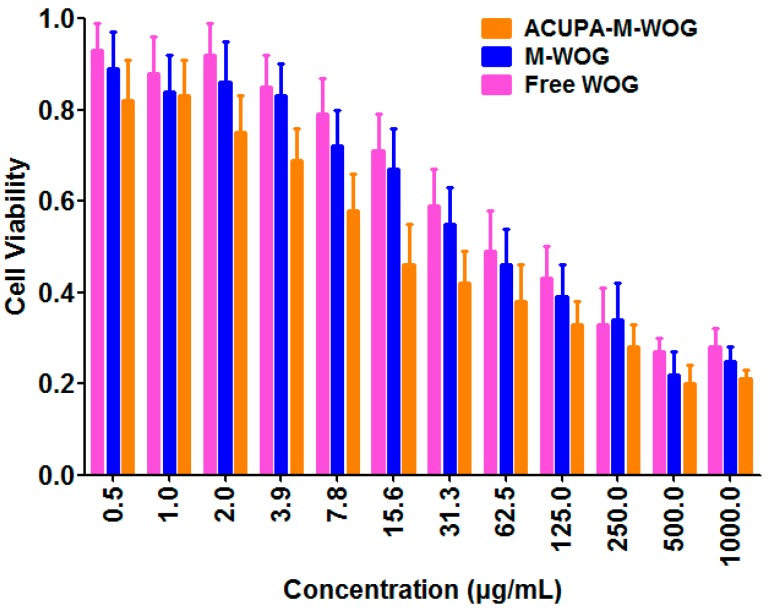
The cytotoxicity of the free WOG, M-WOG, and ACUPA-M-WOG on LNCaP *in vitro*. The cell viability is quantified by MTT through detecting the percentage of viable cells. Mean values and 95% confidence intervals are shown, which are depended on three independent experiments.

**Figure 6:**
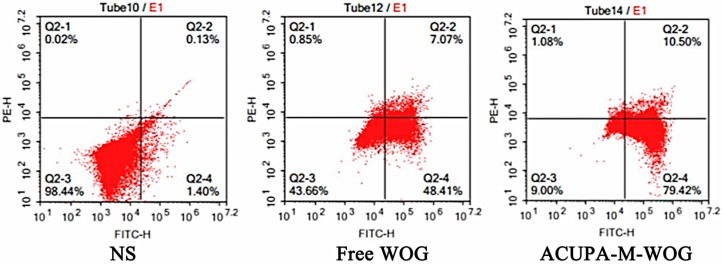
The apoptosis of PC-3 cells detected by Annexin V-FITC/PI stain. After treatment with normal saline (NS), free WOG (10 µM) or Chol-PEG-DUP1-M-WOG (10 µM), the PC-3 cells are stained with Annexin V-FITC/PI and the apoptosis is detected by flow cytometry.

**Figure 7:**
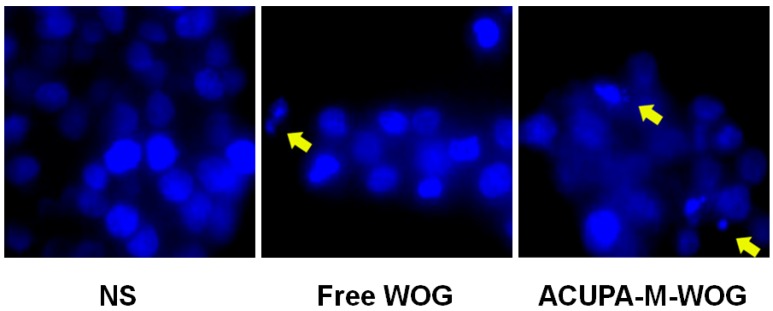
Fluorescent microscopic image. The yellow arrows mean the apoptotic cells. 40× objective lens.

The cellular proliferation assay of ACUPA-M-WOG, M-WOG, blank micelles and free WOG is determined by methyl thiazolyl tetrazolium (MTT) on LNCaP and PC-3 cells, whose results are revealed in Figure 5. After incubated 48 h with the free WOG, M-WOG or ACUPA-M-WOG, the cell survival ratios are detected by MTT at 570 nm. The cell survival ratios are decreased according to the increase of WOG concentration, and there are significant differences in the ACUPA-M-WOG, M-WOG and free WOG groups. Moreover, with the increase of ACUPA-PEG-Chol, there is no significant cell proliferation inhibition observed. As shown in Figure 5, the mean concentrations of wogonin that cause 50% cell inhibition (IC_50_) of ACUPA-M-WOG and M-WOG are respectively 15.83 and 45.65 µg/mL, while that of free wogonin is 49.31 µg/mL. Additionally, the cytotoxicity of ACUPA-M-WOG and M-WOG are not obviously different from free WOG on the PC-3 cells, and there are no significant differences between ACUPA-M-WOG group and M-WOG group, which further proves the surface ACUPA modifications of ACUPA-M-WOG can achieve PCa targeting via PSMA-positive cells. The *in vitro* apoptosis assay is conducted using flow cytometry by Annexin V/PI staining. After incubation with ACUPA-M-WOG, M-WOG or free WOG for 48 h, both Annexin V^+^/PI^−^ and Annexin V^+^/PI^+^ cells are detected and numbered. As showed in Figure 6, there are 89.92% ± 5.30% of apoptotic cells in the ACUPA-M-WOG group, which is markedly higher than in free WOG (55.48% ± 4.89%, *p* < 0.05), and NS (1.53% ± 1.02%, *p* < 0.01) groups. The percentages of Annexin V^+^/PI^+^ cells between ACUPA-M-WOG and free WOG groups are no different, and are respectively 10.50% ± 2.71% and 7.07% ± 2.59%. Meanwhile, Annexin V^+^/PI^−^ cells in the ACUPA-M-WOG group (79.42% ± 4.24%) are much more prevalent than in the free WOG group (48.41% ± 3.05%, *p* < 0.05). Meanwhile, the morphological observation demonstrates that ACUPA-M-WOG can induce stronger cellular apoptosis than free WOG and M-WOG in LNCaP cells, which may come from the uptake difference of each group (Figure 7).

### 2.4. Western Blot Analysis

**Figure 8:**
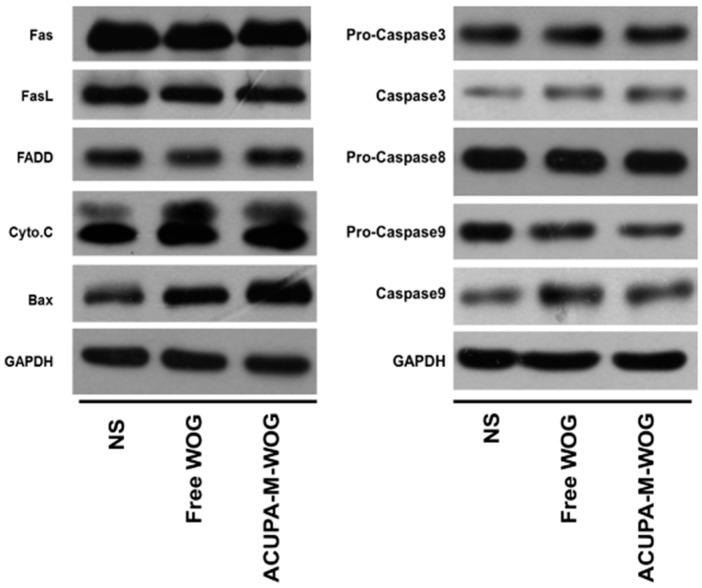
Free WOG or ACUPA-M-WOG induces apoptosis through the intrinsic pathway in PSMA (+) LNCaP cells, which is verified by WB (Western blot). GAPDH (glyceraldehyde phosphate dehydrogenase) served as a control.

**Figure 9:**
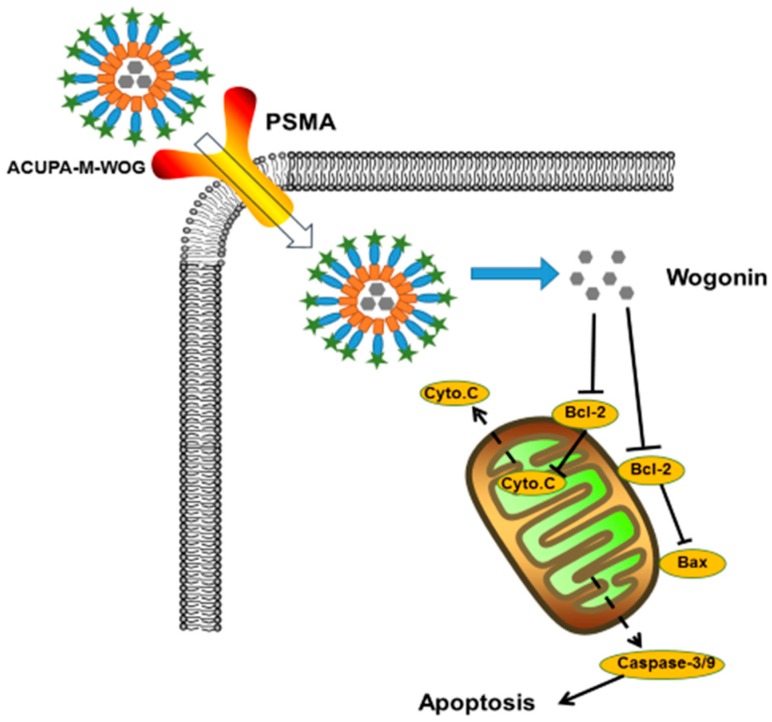
Potential mechanism of ACUPA-M-WOG is dependent on the regulating intrinsic apoptosis. The solid line means that there is direct interaction between the two molecules, while the dash line means that the interaction is indirect.

After incubated with ACUPA-M-WOG or free WOG, the proteins in LNCaP cells are collected, and used to evaluate the levels of apoptosis related proteins by immunoblotting assays. Besides the mitochondrial related apoptotic proteins (intrinsic pathway), the proteins participated in the death receptor pathway (extrinsic pathway) are mainly concerned. The extrinsic pathway can be activated by the ligands of the death receptor super-family, such as TRAIL, IFN-γ, Fas, or TNF-α. On the other hand, the mitochondrial pathway involves many apoptotic related proteins, for example apoptosis inducing factor (AIF), caspase-3, caspase-9, and the Bcl-2 family members. The initial mechanistic studies show that there are no obvious expression level changes of FasL, FADD, and caspase-8 between ACUPA-M-WOG, free WOG and control groups (Figure 8). These results suggest that the apoptotic manner induced by ACUPA-M-WOG rarely involves the extrinsic pathway. On the other hand, ACUPA-M-WOG and free WOG increase the levels of Bax and cytochrome c (Figure 8), suggesting that WOG and ACUPA-M-WOG induced apoptosis in a manner partly dependent on the intrinsic pathway. Based on the fact that cytochrome c released from mitochondria can induce the activation of the proapoptotic caspase cascade, we next investigate the status of caspases 3 and 9. WOG or ACUPA-M-WOG can activate caspases 3 and 9 in LNCaP cells, and eliminate the level of original caspase 9 proteins (Figure 8). These data provide evidence that WOG-induced apoptosis primarily depends on the mitochondrial pathway. Moreover, ACUPA-M-WOG can enhance the apoptosis-inducing ability of WOG for PSMA-positive PCa cells (Figure 9).

## 3. Materials and Methods

### 3.1. Materials and Cell Lines

The 3-[4,5-dimethylthiazol-2-yl]-2,5-diphenltetrazolium bromide (MTT), 1-(3-dimethylaminopropyl)-3-ethylcarbodiimide hydrochloride (EDC), polyethylene glyco (PEG, *M*_W_ = 2000) and Cholesterol (Chol) were purchased from BoAo Biological Technology (Shanghai, China). Succinic anhydride (suc) and coumarin-6 were purchased from Sigma-Aldrich (St. Louis, MO, USA). The N-protected amino acids were obtained from Chengdu Kaijie Co., Ltd. (Chengdu, China). Wogonin (WOG) was obtained from Energy Chem. Co., Ltd. (Shanghai, China). The ultrapure water was produced by a Milli-Q water system. The LNCaP and PC-3 cells were purchased from the American Type Culture Collection (ATCC, Rockville, MD, USA). LNCaP and PC-3 cells were cultured in Dulbecco’s modified eagle medium (DMEM, Gibco, Grand Island, NY, USA) and Roswell Park Memorial Institute 1640 medium (RPMI 1640, Gibco), respectively, and supplemented with 10% fetal bovine serum (FBS, Gibco). The cells were cultured at 37 °C in a humidified incubator, in which the concentration of CO_2_ was 5%.

### 3.2. Synthesis and Characterization of ACUPA-PEG-Cholesterol Conjugates

**Scheme 1:**
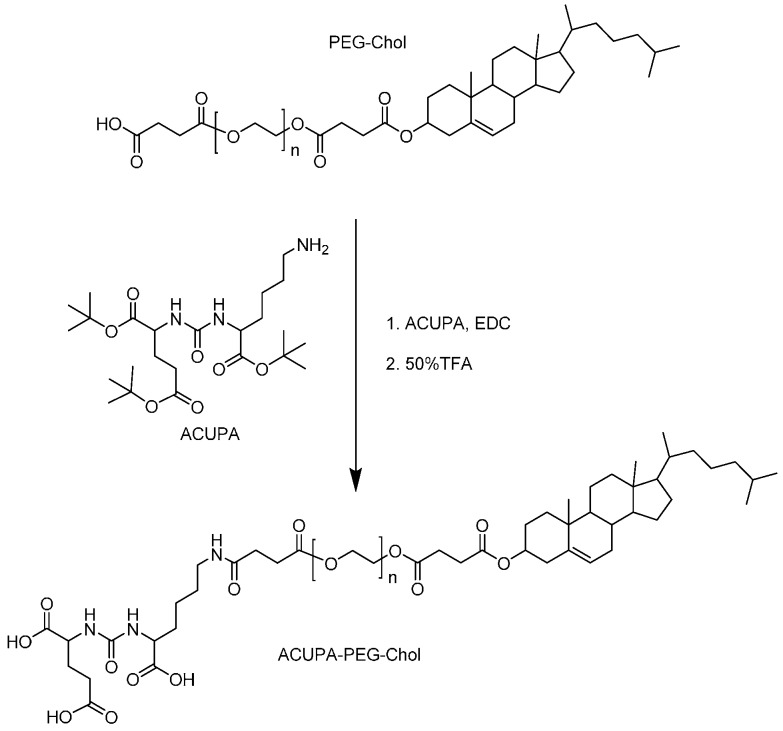
Synthetic route of ACUPA-PEG-Chol.

The synthesis of ACUPA and cholesterol-poly (ethylene glycol)-ACUPA conjugates (ACUPA-PEG-Chol) followed the literature’s method with some modifications [56,61,62,63,64,65,66], and the synthetic route was described in Scheme 1. Briefly, the succinate Chol-PEG (2.0 g, 0.86 mmol) and *tert*-butyl ester of ACUPA (0.43 g, 0.92 mmol) were added into in 20 mL of dichloromethane, and EDC (0.24 g, 0.92 mmol) was dissolved and further stirred for 24 h at 25 °C. Then the *tert*-butyl ester groups of ACUPA were cleared away by 50% TFA (trifluoroacetic acid) and continuous stirred 12 to 24 h. When the reaction was finished, the liquid was dialyzed (membrane tubing, molecular weight cut off = 1.0 kDa) and lyophilized and the final products ACUPA-PEG-Chol (0.78 g, 32.7%) were obtained.

### 3.3. Preparation and Characterization of WOG Loaded ACUPA-PEG-Cholesterol Micelles

The wogonin loaded non-targeted micelles (M-WOG) and ACUPA modified micelles (ACUPA-M-WOG) were prepared as following: WOG and copolymer (1:19, *w*/*w*) were fully mixed in ethanol, and then the mixture was treated with a rotary evaporator under vacuum for 15 min to remove the liquid. The residual material was mixed with normal saline (NS), and then the amphiphilic polymer was re-dissolved and self-assembled to form M-WOG and ACUPA-M-WOG, respectively. The obtained drug loaded micelles was filtered by a 0.22 µm filter (Millipore Co., Waltham, MA, USA) and lyophilized into powder. To determine the particle size distribution and ζ potential of micelles, a laser particle size analyzer (Malvern Nano ZS-90, Worcestershire, UK) was applied to the ACUPA-M-WOG. All the results were verified in three independent samples, and datum were showed as the mean ± standard deviation (SD). The transmission electron microscope (TEM, H-6009IV, Hitachi, Tokyo, Japan) was used in the morphological characterization of ACUPA-M-WOG, all test samples were stained by phosphotungstic acid. The determination of the drug loading (DL) and encapsulation efficiency (EE) were performed on a high performance liquid chromatography instrument (HPLC, Shimadzu LC-20A, Kyoto, Japan) containing a PDA (Photo-Diode Array) detector and a reversed phase C18 column (4.6 × 150 mm, 5 µm, Inertsil/WondaSil, Shimadzu-GL, Kyoto, Japan). The DL and EE of ACUPA-M-WOG were counted via the following equations:

DL = Drug/(Drug + Polymer) × 100%(1)

EE = Drug in micelles/drug in feed × 100%(2)

### 3.4. Cellular Proliferation Inhibition and Uptake of the Micelles

The cellular proliferation activities of ACUPA-M-WOG, M-WOG and free WOG on LNCaP and PC-3 cells were detected by MTT assay, respectively. The cancer cells were incubated in 96-well plates, along with ACUPA-M-WOG, M-WOG or free WOG with a concentration gradient for 48 h, respectively. After three independent experiments, the cell proliferation ratios of all groups were measured. All the data were shown as mean ± SD.

The LNCaP or PC-3 cells (5 × 10^4^ cells per well) were seeded in the six-well plate (Corning, NY, USA) and further cultured 48 h in a humidified incubator. Then, cells were respectively treated with the conventional micelles containing coumarine-6 or ACUPA modified micelles (the concentration of coumarine-6 was 40 ng/mL). After being further incubated for one hour, the culture medium was discarded, and the cells were digested with trypsin. Then the cells were re-suspended with PBS after washed twice. Finally, all the samples were analyzed via flow cytometry (EPICS Elite ESP, Beckman Coulter, Brea, CA, USA) to qualify the fluorescence intensity of coumarin-6.

### 3.5. Apoptosis Assay by Flow Cytometry

The apoptosis-inducing activities of ACUPA-M-WOG, M-WOG and free WOG to LNCaP cells were tested on a flow cytometry. Briefly, LNCaP cells were cultured in 6-well plates for 24–48 h, and then the cells were exposed to DMEM culture containing ACUPA-M-WOG, M-WOG or free WOG for an added 48 h, respectively. Then, cells were digested with trypsin, washed by PBS and stained with Annexin V/Propidium Iodide (BD PharMingen, San Diego, CA, USA). Both early apoptotic and late apoptotic cells were counted by flow cytometry in cell apoptosis determinations. Annexin V^+^/PI^−^ cells meant early apoptotic cells, while Annexin V^+^/PI^+^ cells meant late apoptotic cells.

### 3.6. Western Blot Analysis

The process of protein extraction was finished at 4 °C. First, the total proteins of ACUPA-M-WOG or free WOG treated LNCaP cells were extracted by RIPA buffer (SolarBio, Beijing, China), which contained 1% (*v*/*v*) PMSF (SolarBio), 0.3% (*v*/*v*) protease inhibitor (Sigma, St. Louis, MO, USA) and 0.1% (*v*/*v*) phosphorylated proteinase inhibitor (Sigma). Then, after being centrifuged at 12,000 rpm for 10 min, all the supernatant of lysates was collected. At last, the protein concentration was qualified by the BCA protein assay kit (Pierce, Waltham, MA, USA). SDS-PAGE gel electrophoresis was performed to separate the total protein, which was then transferred onto PVDF membranes. Further treatment with skimmed milk or bovine serum at room temperature was performed to block the non-specfic interactions. The primary antibodies were incubated with PVDF membranes overnight at 4 °C or two hours at room temperature. After being washed several times, the PVDF membranes were incubated in HRP-conjugated IgG (Abmart, Shanghai, China) for several hours. The target proteins were detected through an enhanced chemiluminescence (Millipore, Billerica, MA, USA) based on the manufacturer’s protocol.

## 4. Conclusions

In conclusion, PSMA-positive PCa-targeted polymeric micelles, which are modified with ACUPA on the surface, have been an effective strategy for targeted chemotherapy. The prepared ACUPA-M-WOG not only has small particle size, but also good physico-chemical stability. *In vitro* experiments suggest that compared to a free drug or blank micelle, ACUPA-M-WOG shows stronger cytotoxicity *in vitro* proliferation assays and induces more apoptosis in PSMA-positive PCa cell lines. Moreover, flow cytometry analysis indicates that the surface ACUPA fragment of ACUPA-M-WOG promotes the selective uptake by LNCaP cells which are PSMA positive. Furthermore, the mechanism study of ACUPA-M-WOG demonstrates that the targeted micelles enhance the therapeutic efficiency of WOG, mainly in a manner dependent on inducing intrinsic apoptosis. Based on these, ACUPA-M-WOG may be an effective strategy for a prostate-cancer-targeted drug delivery system.

## References

[1] Mapelli P., Picchio M. (2015). Initial prostate cancer diagnosis and disease staging-the role of choline-PET-CT. Nat. Rev. Urol..

[2] Fernandez-Garcia E.M., Vera-Badillo F.E., Perez-Valderrama B., Matos-Pita A.S., Duran I. (2015). Immunotherapy in prostate cancer: Review of the current evidence. Clin. Transl. Oncol..

[3] Svenson S. (2012). Clinical translation of nanomedicines. Curr. Opin. Solid State Mater. Sci..

[4] Song X.R., Cai Z., Zheng Y., He G., Cui F.Y., Gong D.Q., Hou S.X., Xiong S.J., Lei X.J., Wei Y.Q. (2009). Reversion of multidrug resistance by co-encapsulation of vincristine and verapamil in PLGA nanoparticles. Eur. J. Pharm. Sci..

[5] Song X.R., Zheng Y., He G., Yang L., Luo Y.F., He Z.Y., Li S.Z., Li J.M., Yu S., Luo X. (2010). Development of PLGA nanoparticles simultaneously loaded with vincristine and verapamil for treatment of hepatocellular carcinoma. J. Pharm. Sci..

[6] Ma Q., Li B., Yu Y.Y., Zhang Y., Wu Y., Ren W., Zheng Y., He J., Xie Y.M., Song X.R. (2013). Development of a novel biocompatible poly(ethylene glycol)-block-poly(γ-cholesterol-l-glutamate) as hydrophobic drug carrier. Int. J. Pharm..

[7] Lee D.H., Rhee J.G., Lee Y.J. (2009). Reactive oxygen species up-regulate p53 and Puma; a possible mechanism for apoptosis during combined treatment with TRAIL and wogonin. Br. J. Pharmacol..

[8] Lee D.H., Kim C., Zhang L., Lee Y.J. (2008). Role of p53, Puma, and Bax in wogonin-induced apoptosis in human cancer cells. Biochem. Pharmacol..

[9] Yo J.R., Kim K.J. (1991). Energy transfer from copolymers of styrene-maleic acid to Eu(III) ions in tetrahydrofuran. Anal. Sci..

[10] Murphy G., Ragde H., Kenny G., Barren R., Erickson S., Tjoa B., Boynton A., Holmes E., Gilbaugh J., Douglas T. (1995). Comparison of prostate-specific membrane antigen, and prostate-specific antigen levels in prostatic-cancer patients. Anticancer Res..

[11] Heston W.D.W. (1996). Potential uses of prostate specific membrane antigen (PSMA): A neurocarboxypeptidase and membrane folate hydrolase. Urologe A.

[12] Holmes E.H., Greene T.G., Tino W.T., Boynton A.L., Aldape H.C., Misrock S.L., Murphy G.P. (1996). Analysis of glycosylation of prostate-specific membrane antigen derived from LNCaP cells, prostatic carcinoma tumors, and serum from prostate cancer patients. Prostate.

[13] Murphy G.P., Barren R.J., Erickson S.J., Bowes V.A., Wolfert R.L., Bartsch G., Klocker H., Pointner J., Reissigl A., McLeod D.G. (1996). Evaluation and comparison of two new prostate carcinoma markers—Free-prostate specific antigen and prostate specific membrane antigen. Cancer.

[14] Tsui F.J., McGartland L.P., Ignatoff J.M., Kaul K.L. (1996). Detection of PSA- and PSMA-expressing cells in archived pelvic lymph nodes by RT-PCR. Am. J. Pathol..

[15] Dumas F., Gala J.L., Berteau P., Brasseur F., Eschwege P., Paradis V., Lacour B., Philippe M., Loric S. (1999). Molecular expression of PSMA mRNA and protein in primary renal tumors. Int. J. Cancer.

[16] Luthi-Carter R., Barczak A.K., Speno H., Coyle J.T. (1998). Molecular characterization of human brain *N*-acetylated α-linked acidic dipeptidase (NAALADase). J. Pharmacol. Exp. Ther..

[17] Maurer T., Weirich G., Schottelius M., Weineisen M., Frisch B., Okur A., Kubler H., Thalgott M., Navab N., Schwaiger M. (2015). Prostate-specific membrane antigen-radioguided surgery for metastatic lymph nodes in prostate cancer. Eur. Urol..

[18] Tsourlakis M.C., Klein F., Kluth M., Quaas A., Graefen M., Haese A., Simon R., Sauter G., Schlomm T., Minner S. (2015). PSMA expression is highly homogenous in primary prostate cancer. Appl. Immunohistochem. Mol. Morphol..

[19] Wang H.L., Wang S.S., Song W.H., Pan Y., Yu H.P., Si T.G., Liu Y., Cui X.N., Guo Z. (2015). Expression of prostate-specific membrane antigen in lung cancer cells and tumor neovasculature endothelial cells and its clinical significance. PLoS ONE.

[20] Zhu Z.Y., Zhong C.P., Xu W.F., Lin G.M., Ye G.Q.W., Ji Y.Y., Sun B., Yeh M. (1999). PSMA mimotope isolated from phage displayed peptide library can induce PSMA specific immune response. Cell Res..

[21] Aggarwal S., Singh P., Topaloglu O., Isaacs J.T., Denmeade S.R. (2006). A dimeric peptide that binds selectively to prostate-specific membrane antigen and inhibits its enzymatic activity. Cancer Res..

[22] Shen D.W., Xie F., Edwards W.B. (2013). Evaluation of phage display discovered peptides as ligands for prostate-specific membrane antigen (PSMA). PLoS ONE.

[23] Lupold S.E., Hicke B.J., Lin Y., Coffey D.S. (2002). Identification and characterization of nuclease-stabilized RNA molecules that bind human prostate cancer cells via the prostate-specific membrane antigen. Cancer Res..

[24] DeWeese T.L., Ni X., Zhang Y., Lupold S. (2009). PSMA aptamer-targeted sirnas selectively enhance prostate cancer radiation sensitivity. Int. J. Radiat. Oncol..

[25] DeWeese T.L., Ni X., Zhang Y., Hedayati M., Lupold S. (2010). Substantial increases in prostate cancer tumor control result from a combination of PSMA aptamer-targeted siRNAs and radiation. Int. J. Radiat. Oncol..

[26] Ni X.H., Zhang Y.G., Ribas J., Chowdhury W.H., Castanares M., Zhang Z.W., Laiho M., DeWeese T.L., Lupold S.E. (2011). Prostate-targeted radiosensitization via aptamer-shRNA chimeras in human tumor xenografts. J. Clin. Investig..

[27] Ni X.H., Zhang Y.G., Zennami K., Castanares M., Mukherjee A., Raval R.R., Zhou H.M., DeWeese T.L., Lupold S.E. (2015). Systemic administration and targeted radiosensitization via chemically synthetic aptamer-siRNA chimeras in human tumor xenografts. Mol. Cancer Ther..

[28] Chang S.S., Reuter V.E., Heston W.D.W., Bander N.H., Grauer L.S., Gaudin P.B. (1999). Five different anti-prostate-specific membrane antigen (PSMA) antibodies confirm PSMA expression in tumor-associated neovasculature. Cancer Res..

[29] Sanna V., Pala N., Sechi M. (2014). Targeted therapy using nanotechnology: Focus on cancer. Int. J. Nanomed..

[30] Tykvart J., Navratil V., Sedlak F., Corey E., Colombatti M., Fracasso G., Koukolik F., Barinka C., Sacha P., Konvalinka J. (2014). Comparative analysis of monoclonal antibodies against prostate-specific membrane antigen (PSMA). Prostate.

[31] Zuccolotto G., Fracasso G., Merlo A., Montagner I.M., Rondina M., Bobisse S., Figini M., Cingarlini S., Colombatti M., Zanovello P. (2014). PSMA-specific CAR-engineered T cells eradicate disseminated prostate cancer in preclinical models. PLoS ONE.

[32] Morris M.J., Divgi C.R., Pandit-Taskar N., Batraki M., Warren N., Nacca A., Smith-Jones P., Schwartz L., Kelly W.K., Slovin S. (2005). Pilot trial of unlabeled and indium-111-labeled anti-prostate-specific membrane antigen antibody J591 for castrate metastatic prostate cancer. Clin. Cancer Res..

[33] Osborne J.R., Akhtar N.H., Vallabhajosula S., Anand A., Deh K., Tagawa S.T. (2013). Prostate-specific membrane antigen-based imaging. Urol. Oncol.-Semin. Orig..

[34] Barren R.J., Holmes E.H., Boynton A.L., Misrock S.L., Murphy G.P. (1997). Monoclonal antibody 7e11.C5 staining of viable LNCaP cells. Prostate.

[35] Smith-Jones P.M., Vallabhajosula S., Navarro V., Bastidas D., Goldsmith S.J., Bander N.H. (2003). Radiolabeled monoclonal antibodies specific to the extracellular domain of prostate-specific membrane antigen: Preclinical studies in nude mice bearing LNCaP human prostate tumor. J. Nucl. Med..

[36] Ruggiero A., Holland J.P., Hudolin T., Shenker L., Koulova A., Bander N.H., Lewis J.S., Grimm J. (2011). Targeting the internal epitope of prostate-specific membrane antigen with Zr-89-7E11 immuno-PET. J. Nucl. Med..

[37] Ma D.S., Hopf C.E., Malewicz A.D., Donovan G.P., Senter P.D., Goeckeler W.F., Maddon P.J., Olson W.C. (2006). Potent antitumor activity of an auristatin-conjugated, fully human monoclonal antibody to prostate-specific membrane antigen. Clin. Cancer Res..

[38] Di Pippo V.A., Olson W.C., Nguyen H.M., Brown L.G., Vessella R.L., Corey E. (2015). Efficacy studies of an antibody-drug conjugate PSMA-ADC in patient-derived prostate cancer xenografts. Prostate.

[39] Petrylak D.P., Kantoff P.W., Mega A.E., Vogelzang N.J., Stephenson J., Fleming M.T., Stambler N., Petrini M., Huang K., Israel R.J. (2013). Prostate-specific membrane antigen antibody drug conjugate (PSMA ADC): A phase I trial in metastatic castration-resistant prostate cancer (mCRPC) previously treated with a taxane. J. Clin. Oncol..

[40] Petrylak D., Kantoff P., Mega A., Stephenson J., Vogelzang N., Fleming M., Blattman S., Stambler N., D’Ambrosio P., Israel R.J. (2012). Prostate specific membrane antigen antibody drug conjugate (PSMA ADC): A phase 1 trial in castration-resistant metastatic prostate cancer (mCRPC). Eur. J. Cancer.

[41] Petrylak D.P., Smith D.C., Appleman L.J., Fleming M.T., Hussain A., Dreicer R., Sartor A.O., Shore N.D., Vogelzang N.J., Youssoufian H. (2014). A phase 2 trial of prostate-specific membrane antigen antibody drug conjugate (PSMA ADC) in taxane-refractory metastatic castration-resistant prostate cancer (mCRPC). J. Clin. Oncol..

[42] Tolcher A., Ejadi S., Sartor A.O., Nguyen B., Habbe A., Vogelzang N. (2015). A ph 1 study of 2 different schedules of the PSMA-tubulysin small-molecule drug conjugate EC1169 in pts with rec Met Cast-Resist PC (mCRPC). Ann. Oncol..

[43] Weineisen M., Schottelius M., Simecek J., Baum R.P., Yildiz A., Beykan S., Kulkarni H.R., Lassmann M., Klette I., Eiber M. (2015). Ga-68- and lu-177-labeled PSMA I&T: Optimization of a PSMA-targeted theranostic concept and first proof-of-concept human studies. J. Nucl. Med..

[44] Azad B.B., Banerjee S.R., Pullambhatla M., Lacerda S., Foss C.A., Wang Y.C., Ivkov R., Pomper M.G. (2015). Evaluation of a PSMA-targeted bnf nanoparticle construct. Nanoscale.

[45] Tse B.W.C., Cowin G.J., Soekmadji C., Jovanovic L., Vasireddy R.S., Ling M.T., Khatri A., Liu T.Q., Thierry B., Russell P.J. (2015). PSMA-targeting iron oxide magnetic nanoparticles enhance MRI of preclinical prostate cancer. Nanomedicine.

[46] Zhang L., Jiang Y.P., Li X.G., Wang S.R., Xiao Y. (2015). Preparation of titanium dioxide nanoparticles modified with methacrylate and their electrophoretic properties. J. Mater. Sci.-Mater. Electron..

[47] Xiang B., Dong D.W., Shi N.Q., Gao W., Yang Z.Z., Cui Y., Cao D.Y., Qi X.R. (2013). PSA-responsive and PSMA-mediated multifunctional liposomes for targeted therapy of prostate cancer. Biomaterials.

[48] Baek S.E., Lee K.H., Park Y.S., Oh D.K., Oh S., Kim K.S., Kim D.E. (2014). RNA aptamer-conjugated liposome as an efficient anticancer drug delivery vehicle targeting cancer cells *in vivo*. J. Control. Release.

[49] Park H., Kim D.M., Baek S.E., Kim K.S., Kim D.E. (2015). Comparison of drug delivery efficiency between doxorubicin intercalated in RNA aptamer and one encapsulated in RNA aptamer-conjugated liposome. Bull. Korean Chem. Soc..

[50] Kularatne S.A., Wang K., Santhapuram H.K.R., Low P.S. (2009). Prostate-specific membrane antigen targeted imaging and therapy of prostate cancer using a PSMA inhibitor as a homing ligand. Mol. Pharm..

[51] Peng Z.H., Sima M., Salama M.E., Kopeckova P., Kopecek J. (2013). Spacer length impacts the efficacy of targeted docetaxel conjugates in prostate-specific membrane antigen expressing prostate cancer. J. Drug Target..

[52] Bandari R.P., Jiang Z.R., Reynolds T.S., Bernskoetter N.E., Szczodroski A.F., Bassuner K.J., Kirkpatrick D.L., Rold T.L., Sieckman G.L., Hoffman T.J. (2014). Synthesis and biological evaluation of copper-64 radiolabeled [DUPA-6-Ahx-(NODAGA)-5-Ava-BBN(7-14)NH_2_], a novel bivalent targeting vector having affinity for two distinct biomarkers (GRPr/PSMA) of prostate cancer. Nucl. Med. Biol..

[53] Winter G., Baur B., Andreolli E., Kull T., Witulla B., Solbach C., Machulla H. (2013). Biological evaluation of the new glutamate-urea-based PSMA ligand Df-DUPA-Pep. Eur. J. Nucl. Med. Mol. Imaging.

[54] Baur B., Andreolli E., Al-Momani E., Malik N., Machulla H.J., Reske S.N., Solbach C. (2014). Synthesis and labelling of Df-DUPA-Pep with gallium-68 and zirconium-89 as new PSMA ligands. J. Radioanal. Nucl. Chem..

[55] Baranyai Z., Reich D., Vagner A., Weineisen M., Toth I., Wester H.J., Notni J. (2015). A shortcut to high-affinity Ga-68 and Cu-64 radiopharmaceuticals: One-pot click chemistry trimerisation on the trap platform. Dalton Trans..

[56] Roy J., Nguyen T.X., Kanduluru A.K., Venkatesh C., Lv W., Reddy P.V.N., Low P.S., Cushman M. (2015). DUPA conjugation of a cytotoxic indenoisoquinoline topoisomerase i inhibitor for selective prostate cancer cell targeting. J. Med. Chem..

[57] Chen X.A., Wang X.H., Wang Y.S., Yang L., Hu J., Xiao W.J., Fu A., Cai L.L., Li X., Ye X. (2010). Improved tumor-targeting drug delivery and therapeutic efficacy by cationic liposome modified with truncated bFGF peptide. J. Control. Release.

[58] Cai L.L., Qiu N., Li X., Luo K.L., Chen X., Yang L., He G., Wei Y.Q., Chen L.J. (2011). A novel truncated basic fibroblast growth factor fragment-conjugated poly (ethylene glycol)-cholesterol amphiphilic polymeric drug delivery system for targeting to the FGFR-overexpressing tumor cells. Int. J. Pharm..

[59] Wang X.H., Deng L.Y., Chen X.A., Pei H.Y., Cai L.L., Zhao X., Wei Y.Q., Chen L.J. (2011). Truncated bFGF-mediated cationic liposomal paclitaxel for tumor-targeted drug delivery: Improved pharmacokinetics and biodistribution in tumor-bearing mice. J. Pharm. Sci..

[60] Zhao Y.Z., Li X., Lu C.T., Lin M., Chen L.J., Xiang Q., Zhang M., Jin R.R., Jiang X., Shen X.T. (2014). Gelatin nanostructured lipid carriers-mediated intranasal delivery of basic fibroblast growth factor enhances functional recovery in hemiparkinsonian rats. Nanomedicine.

[61] Zhao Y.B., Lin D.Y., Wu F.B., Guo L., He G., Ouyang L., Song X.R., Huang W., Li X. (2014). Discovery and *in vivo* evaluation of novel RGD-modified lipid-polymer hybrid nanoparticles for targeted drug delivery. Int. J. Mol. Sci..

[62] Yu Y.Y., He Y.J., Xu B., He Z.Y., Zhang Y., Chen Y., Yang Y., Xie Y.M., Zheng Y., He G. (2013). Self-assembled methoxy poly(ethylene glycol)cholesterol micelles for hydrophobic drug delivery. J. Pharm. Sci..

[63] Wu F.B., Xu T., Liu C., Chen C., Song X.R., Zheng Y., He G. (2013). Glycyrrhetinic acid-poly(ethyleneglycol)-glycyrrhetinic acid tri-block conjugates based self-assembled micelles for hepatic targeted delivery of poorly water soluble drug. Sci. World J..

[64] Kularatne S.A., Venkatesh C., Santhapuram H.K.R., Wang K., Vaitilingam B., Henne W.A., Low P.S. (2010). Synthesis and biological analysis of prostate-specific membrane antigen-targeted anticancer prodrugs. J. Med. Chem..

[65] Banerjee S.R., Pullambhatla M., Foss C.A., Falk A., Byun Y., Nimmagadda S., Mease R.C., Pomper M.G. (2013). Effect of chelators on the pharmacokinetics of *T*c-99m-labeled imaging agents for the prostate-specific membrane antigen (PSMA). J. Med. Chem..

[66] Tykvart J., Schimer J., Jancarik A., Barinkova J., Navratil V., Starkova J., Sramkova K., Konvalinka J., Majer P., Sacha P. (2015). Design of highly potent urea-based, exosite-binding inhibitors selective for glutamate carboxypeptidase ii. J. Med. Chem..

